# Case report: Novel use of the conventional method- chemical nail avulsion may be effective for treatment of green nail syndrome

**DOI:** 10.3389/fmed.2022.991918

**Published:** 2022-08-25

**Authors:** Qian Yu, Yuanyuan Wang, Hong Yang, Wei Li, Lianjuan Yang

**Affiliations:** ^1^Department of Medical Mycology, Shanghai Dermatology Hospital, Shanghai, China; ^2^Department of Medical Cosmetology, Shanghai Dermatology Hospital, Shanghai, China

**Keywords:** green nail syndrome, *Pseudomonas aeruginosa*, predisposing factors, chemical nail avulsion, urea powder

## Abstract

Green nail syndrome (GNS) is a triad of green discoloration of the nail plate, proximal paronychia, and distal onycholysis. *Pseudomonas aeruginosa* is known to be the most common causative agent; however, there is no unified standard for the diagnosis and treatment of GNS. Thus, treatment is challenging and often refractory. Here, we report three representative cases with different predisposing factors, including trauma-related, occupation-related, and onychosis-related GNS. Patients with GNS accompanied by onycholysis were instructed to undergo chemical nail avulsion combined with topical antibiotics, and favorable curative effects were observed in all cases. Chemical nail avulsion with urea powder as a conventional method may be an effective treatment for GNS and warrants clinical generalization.

## Introduction

Green nail syndrome (GNS), also known as chloronychia, is an infectious disorder characterized by a greenish discoloration of the nail plate (1) most commonly caused by *Pseudomonas aeruginosa* (2). Although ubiquitous in nature, *P. aeruginosa* is unable to colonize dry environments and rarely affects healthy skin (3).

Green nail syndrome has a chronic course and often affects aesthetics. However, to date, there is no unified standard for its diagnosis and treatment. Thus, treatment of GNS is always challenging and often refractory (4). Safer, more effective, and widely applicable treatments are urgently needed. Previous studies have reported various predisposing factors to the condition such as trauma, occupation, onychomycosis (5), and psoriasis (6). It is important to carefully analyze the predisposing factors of GNS to assist in the treatment of patients.

Although GNS has rarely been reported in the literature, it is not uncommon in clinical practice. In our hospital, an average of 200 patients with onychosis seek medical advice per week, among those patients about 1–2 patients with GNS. Herein, we describe three representative cases of GNS accompanied by onycholysis, with different predisposing factors. The patients were treated with chemical nail avulsion, and favorable curative effects were achieved.

## Case description

### Case 1

A 25-year-old woman presented with an asymptomatic partial color change of the left thumbnail, which she noticed immediately after the removal of gel nail polish. The patient had a history of obtaining regular manicures and denied a history of trauma or the nail’s prolonged exposure to moisture.

**FIGURE 1:**
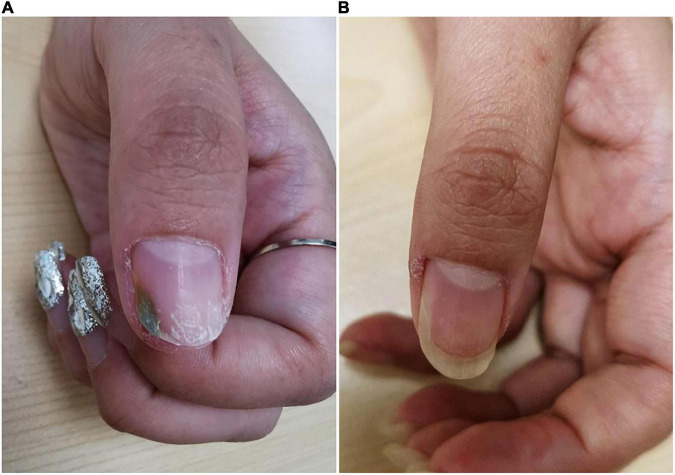
Clinical image of Case 1. **(A)** Nail plate of a patient with trauma-related GNS at initial visit. **(B)** Subsided lesions at 3 months following topical antibiotic treatment.

Physical examination revealed green discoloration of the middle to distal parts of the left thumbnail, accompanied by a slight distal onycholysis (Figure 1A). Direct mycologic examination of scrapings and culture was negative, however, the bacterial culture of the nail scraping was positive for *P. aeruginosa*. A drug susceptibility test with *P. aeruginosa* showed its sensitivity to levofloxacin. The patient was then instructed to remove the gel nail polish and cease manicures. She received chemical nail avulsion with urea powder for 3 days to remove the left distal portions of the thumbnail plate, and subsequently applied topical nadifloxacin twice daily. At 3-month follow-up, the nail discoloration had resolved completely (Figure 1B).

### Case 2

A 36-year-old otherwise healthy man was referred to our hospital in the summer with a 2-month history of green discoloration on his right third fingernail. Discoloration appeared at the distal margin and gradually spread throughout the nail; a similar nail lesion involved the thumbnail. The patient was regularly engaged in garbage sorting and wore gloves daily for extended periods of time.

**FIGURE 2:**
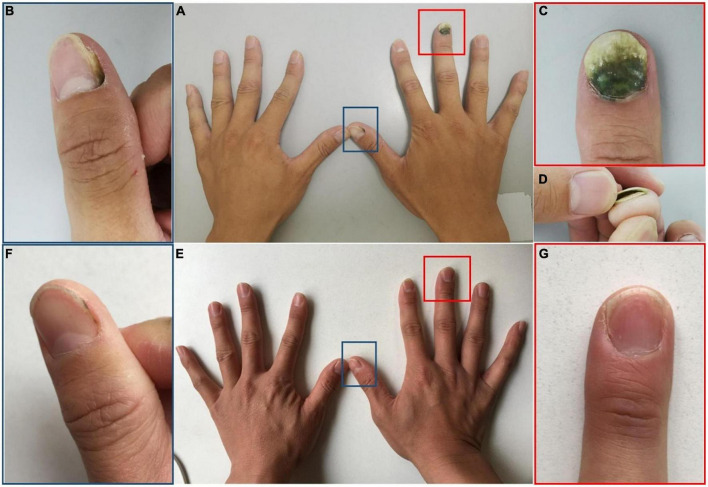
Clinical image of Case 2. **(A–D)** Nail plate of a patient with occupation-related GNS at initial visit. **(E,F)** Subsided lesions at 6 months following chemical nail avulsion combined with topical antibiotic treatment. **(B,C)** Are partial magnifications of **(A)**. **(D)** Is profile image of **(C)**. **(F,G)** Are partial magnifications of **(E)**.

Dermatologic examination showed dark-greenish pigmentation and onycholysis on the right third fingernail (Figures 2A,C,D), green discoloration adjacent to the lateral nail fold on the right thumbnail, and corresponding periungual skin red swelling (Figures 2A,B). Fungal coinfections were excluded by direct fluorescence microscopy and culture. However, bacterial cultures of nail scrapings were positive for *P. aeruginosa*. An *in vitro* drug susceptibility test indicated high sensitivity to levofloxacin. The patient was instructed to stop wearing gloves and keep his hands dry. He was then treated with chemical nail avulsion combined with nadifloxacin, as in Case 1. The nail plates were cured after 6 months of follow-up (Figures 2E–G).

### Case 3

A 57-year-old woman developed an asymptomatic black-greenish discoloration in both thumbnails over the preceding 5 months. The thumbnail plates had gradually appeared yellowish and thickened, and full discoloration had begun 1 month prior to presentation to our hospital. The patient had no significant medical history, had not received any medication, and had not experienced any previous fingernail injuries. As someone with domestic duties, her hands were constantly exposed to water, detergents, and soaps.

**FIGURE 3:**
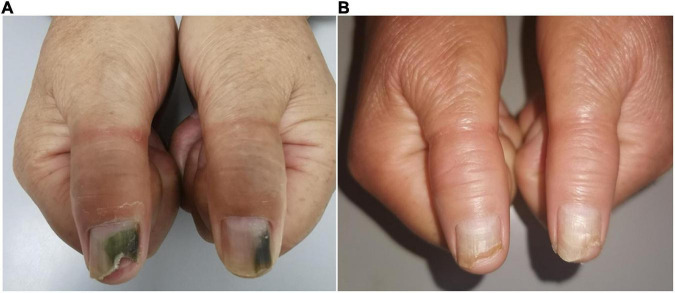
Clinical image of Case 3. **(A)** Nail plate of a patient with onychosis-related GNS at initial visit. **(B)** Improved lesions at 2 months following chemical nail avulsion combined with topical antibiotic treatment.

Clinical examination of the patient’s thumbnails revealed thickened nail plates with green discoloration from the distal edge to the middle nail plates, as well as onycholysis at the site of the discolored area (Figure 3A). Direct mycological examination of nail scrapings showed positivity for fungal hyphae, and cultures were identified as *Trichophyton rubrum*. The bacterial culture was positive for *P. aeruginosa*. A drug susceptibility test showed levofloxacin sensitivity. The patient was instructed to keep the hands dry and treat the primary onychosis. She received treatment with systemic oral itraconazole 400 mg/day for 7 days per month for 3 months along with topical chemical nail avulsion and nadifloxacin. At 2-month follow-up, the nail discoloration had faded significantly. The nail plates had completely healed at 5-month telephone follow-up (Figure 3B).

## Discussion

Green nail syndrome is a triad of green discoloration of the nail plate, proximal paronychia, and distal onycholysis. The characteristic greenish discoloration of the nail results from the antibiotic pigments pyoverdine and pyocyanin produced by *P. aeruginosa*. This bacterium is an opportunistic human pathogen that can cause a wide variety of skin diseases, including GNS, interdigital infection, hot tub folliculitis, and ecthyma gangrenosum (3). Generally, characteristic clinical findings are sufficient for diagnosis of GNS; however, it must be differentiated from onychomycosis caused by dematiaceous fungi, yellow nail syndrome, subungual hematoma, subungual melanoma, exogenous pigmentation, medication-induced effects, and glomus tumor (7). Pathogenic identification, pathological examination, and dermoscopic patterns can be used to confirm intractable GNS.

The pathogenesis of GNS has not been fully elucidated in the literature. However, it is known that when the nail plates undergo barrier impairment or prolonged exposure to a moist environment, *P. aeruginosa* infection is more likely (4). The potential predisposing factors suggested in this study were as follows.

(1) Trauma-related GNS: The predisposing factors for this type of GNS are mainly nail trauma including manicures, wearing tight shoes, and biting or picking nails (8). Nail trauma disrupts the integumentary barrier, making the nail conducive to *P. aeruginosa* invasion. In case 1, the patient obtained regular manicures that involved the cutting, filing, and shaping of the nails; pushing back of cuticles, and application of gel polish onto the nails. These procedures can cause nail plate trauma, enable *P. aeruginosa* colonization, and subsequently create an occlusive environment by covering the nail with gel nail polish, resulting in infection by this pathogen.
(2) Occupation-related GNS: GNS can be viewed as an occupationally triggered disease in those with domestic duties, cleaners, barbers, dishwashers, bakers, and medical personnel (9). These patients usually have a history of long duration of exposure to water or moist conditions and deny a history of nail trauma or previous onychosis. In this report, the patient in Case 2 engaged in garbage sorting and wore gloves for lengthy periods daily, causing hyperhidrosis leading to a moist environment, which is an ideal condition for the growth of *P. aeruginosa*.
(3) Onychosis-related GNS: This type often occurs in patients with prior nail problems, such as onychomycosis, onycholysis, chronic paronychia, and psoriasis. A recent study demonstrated that GNS was identified concurrently with onycholysis in 87% of individuals (10), among which onychomycosis was the most prevalent concomitant disease. Fungal infections create tunnel-like structures in nail keratin, and *P. aeruginosa* can proliferate in these spaces. In Case 3, the patient’s previous onychomycosis contributed to the entry of *P. aeruginosa*; additionally, as someone with domestic duties, her hands often had prolonged exposure to water. These two factors led to the occurrence of GNS.

An optimal treatment regimen for GNS has not yet been established. Topical or systemic antibiotics may be used to eradicate infections, with the application of topical antibiotics showing increased effectiveness in patients due to economic convenience, convenient administration, and few adverse reactions. However, in this drug delivery method, antibiotics may not fully permeate the nail plate to reach the infected area. Removal of the involved nail can massively reduce pathogen load, promote drug permeation, and accelerate disease recovery (11). Currently, chemical nail avulsion with high-concentration urea has gradually superseded surgical avulsion owing to levels of minimal to no pain, low risk of infection, and no risk of hemorrhage. Urea, a keratolytic agent, can disrupt the cell/cell adhesion of corneocytes in the nail plate, and a high concentration can soften the nail plate for easy removal of the infected area without damage to the matrix and nail bed (12, 13). Chemical nail avulsion using urea powder (100% urea without any other ingredients), as a conventional method, has been widely used in the treatment of onychomycosis with a definite therapeutic effect for many years in our hospital (14). Therefore, we assessed this as a treatment for patients with GNS.

**FIGURE 4:**
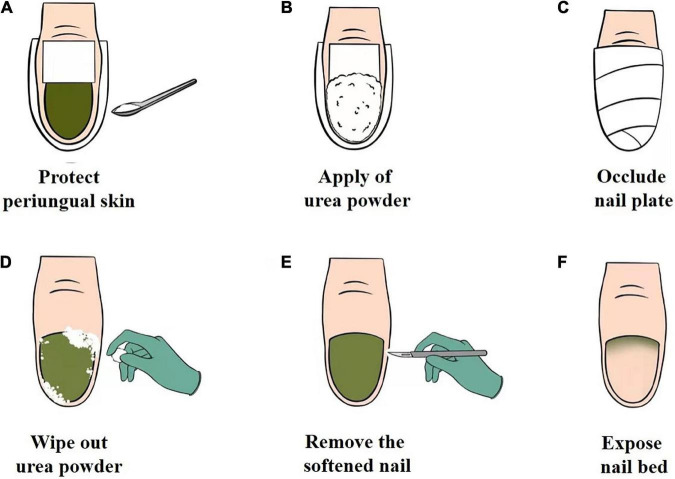
**(A–F)** The operational chemical nail avulsion procedure using urea powder. **(A)** Protection of the periungual skin of the affected nail plate with waterproof plaster. **(B)** Application of urea powder to the affected nail plate. **(C)** Occlusion of the affected nail plate with waterproof plaster. **(D)** Removal of urea powder with an alcohol cotton ball. **(E)** Removal of the softened nail. **(F)** Exposure of the nail bed.

The procedure for chemical nail avulsion with urea powder is shown in Figure 4. The periungual area was protected with a waterproof plaster to prevent chemical irritation of the soft tissue (Figure 4A), followed by the application of urea powder (Figure 4B); and then occluded with a waterproof plaster (Figure 4C). The patient was guided to keep the nail occluded and to avoid wetting the treated area. If the patient were to wet the treated area during chemical nail avulsion, this can lead to urea dissolution. Dissolved urea may then permeate into periungual skin of the treated area and cause skin maceration, slight irritation, or reduction in the efficiency of nail plate removal. After 3–5 days of occlusion, the waterproof plaster was removed, the nail was cleansed with 75% wipes (Figure 4D), and the softened nail was removed using a scalpel (Figures 4E,F). Following this, the patient was instructed to administer topical antibiotics. This approach was able to break the moist, occlusive environment to keep the nail dry and prevent *P. aeruginosa* persistence. This treatment approach softens the infected area of nail plate to achieve its complete removal in an average of 3–5 days, with minimal known adverse reactions. Chemical nail avulsion with urea powder can be applied to all patients, including the elderly and children, as all procedures are performed by podiatry in a hospital setting. In this report, patients who were diagnosed with GNS with concomitant onycholysis underwent chemical nail avulsion combined with topical antibiotics and acquired excellent therapeutic effects. Further studies are required to confirm the efficacy, safety, and tolerability of this treatment.

In conclusion, GNS is a distinct clinical condition that often occurs in patients with various predisposing factors that favor the growth of *P. aeruginosa*. Chemical nail avulsion with urea powder may be an effective method for the treatment of GNS and deserves to be clinically generalized.

## Data availability statement

The raw data supporting the conclusions of this article will be made available by the authors, without undue reservation.

## Ethics statement

The studies involving human participants were reviewed and approved by the Ethics Committee of Shanghai Dermatology Hospital. The patients/participants provided their written informed consent to participate in this study. Written informed consent was obtained from the individual(s) for the publication of any potentially identifiable images or data included in this article.

## Author contributions

QY and YW: design and drafting of the work, analysis, acquisition, and interpretation of data. HY: laboratory testing and data analysis. WL and LY: study design, revision, and finalization of the manuscript. All authors have contributed to the manuscript and approved the submitted version.
